# Supplementary material for: Deuterium-depletion has no significant impact on the mutation rate of Escherichia coli, deuterium abundance therefore has a probabilistic, not deterministic effect on spontaneous mutagenesis
Source: PLoS One. 2021 Mar 8;16(3):e0243517. doi: 10.1371/journal.pone.0243517 (PMC7939293; doi:10.1371/journal.pone.0243517)
Supplement: S1 Fig — (PDF) [file pone.0243517.s001.pdf]

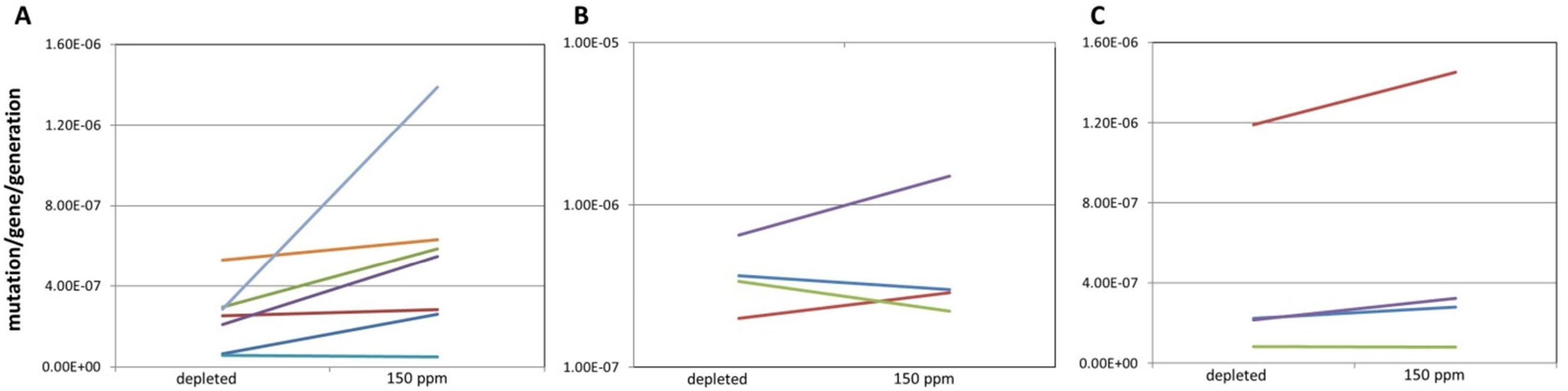

S1 Figure. Effect of D on the mutation rate occurring in *E. coli* cultures. The results of multiple pairwise fluctuation analyses are shown, made in growth media containing <2 ppm or 150 ppm D using three different mutation detection systems, as described in the Materials and Methods. Only those results are shown which passed our filtering. (A) Results obtained using the cycA system. (B) Results obtained using the ackA system. (C) Results obtained using the galK system. The colors representing each pair of experiments corresponds to the colors used on Figure 2.
