# Supplementary material for: Deuterium-depletion has no significant impact on the mutation rate of Escherichia coli, deuterium abundance therefore has a probabilistic, not deterministic effect on spontaneous mutagenesis
Source: PLoS One. 2021 Mar 8;16(3):e0243517. doi: 10.1371/journal.pone.0243517 (PMC7939293; doi:10.1371/journal.pone.0243517)
Supplement: S1 Table — (PDF) [file pone.0243517.s002.pdf]

S1 Table. PCR primers used in this study

**primer name**	**sequence**	**function**
galU_SpF	atgGCTGCCATTAATACGAAAGTCAAAAAAGCCGTTATCCCCTATTTAACGACCCTGCCCTG	generating linear DNA with left homology box
galU_SpR	ttaCTTCTTAATGCCCATCTCTTCTTCAAGCCAGGCTTTAAACGACCGAGTGAGCTGGCTATTTG	generating linear DNA with right homology box
galU_D	TTACCTGCTAATGTCGGCTG	checking, external
galU_E	CGATTGCTCAACGCCGTTTC	checking, external
SmFw	CTTACGTTGTCCCGCATTTGG	checking, internal
