# Supplementary material for: Deuterium-depletion has no significant impact on the mutation rate of Escherichia coli, deuterium abundance therefore has a probabilistic, not deterministic effect on spontaneous mutagenesis
Source: PLoS One. 2021 Mar 8;16(3):e0243517. doi: 10.1371/journal.pone.0243517 (PMC7939293; doi:10.1371/journal.pone.0243517)
Supplement: Attachment — Submitted filename: PlosONEDeuterium ms Review.pdf [file pone.0243517.s006.pdf]

Review

Studies of the impact of D_2O relative to H_2O on biological systems has a long history that has been reviewed recently (Kselikova et al., Folia Microbiologica (2019) 64:673–681); this review cites evidence that unicellular organisms like E. coli tolerate a D_2O environments (This article should be cited). However, it has been demonstrated that the mutagenic activity is enhanced upon incubation of E. coli cells in D_2O. A Double D-Bridge mechanism was proposed to account for these observations; it is based on the hypothesis that the mutagenic activity is proportional to the deuteration of the two A:T hydrogen bonding atoms.

In this work, the mutation rates in three different E. coli genes were determined and interpreted within the framework of the DDB hypothesis. The experimental mutagenicity results and statistical analysis are rigorous and a useful contribution to the sparse literature on this topic.

However, I disagree with the conclusion that there is a disparity between the quantitative predictions of the DDB theory and the author's experimental results. Their apparent and unstated assumption is that every polymerase-catalyzed insertion of the incorrect nucleotide is 100% mutagenic. Polymerase – catalyzed insertions of dNTP nucleotides opposite a template base is a competition between the four nucleotides; the dominant insertion is decided by the differences in equilibrium binding constants of the four dNTPs opposite the nucleotide in the template strand, before the nucleotidyl transfer step occurs. The canonical dNTP usually predominates and the correct nucleotide is inserted.

Deuteration may influence this equilibrium which means that a mutation-inducing event can occur with an efficiency much less than 100%. Since, this efficiency is unknown and can be much smaller than 100%, the DDB mechanism cannot be excluded. In other words, deuteration may not fully inhibit the error-free insertion of the canonical nucleotide which decreases the mutation rate.

In summary, I would recommend publication of this paper if this possibility is properly accounted for in their interpretation, and if they avoid discounting the validity of the DDB hypothesis which is not supported by the data presented (a change in the title and abstract is also called for). Finally, this work is important because it contradicts/corrects previous observations that reported > 1000-fold changes in mutagenicity attributed to deuterium effects which seem improbable.
